# Cultural adaptation of the Work Disability Functional Assessment Battery (WD-FAB) into German (Germany): hybrid application of two adaptation approaches

**DOI:** 10.1186/s41687-026-01181-3

**Published:** 2026-09-08

**Authors:** You-Shan Feng, Sandra Meyer-Moock, Maresa Buchholz, Elizabeth Marfeo, Julia Porcino, Susanne Weinbrenner, Jörg Gehrke, Lewis E. Kazis, Thomas Kohlmann

**Affiliations:** 1 Institute for Clinical Epidemiology and Applied Biostatistics (IKEaB), Universitätsklinikum Tübingen, Tübingen, Germany; 2 Institute for Community Medicine, Greifswald University Hospital, Greifswald, Germany; 3 German Center for Neurodegenerative Diseases (DZNE), Site Rostock/Greifswald, Patient-reported Outcomes & Health Economics Research, Greifswald, Germany; 4 Department of Community Health, Tufts University, Medford, USA; 5 Rehabilitation Medicine Department, National Institutes of Health Clinical Center, Bethesda, USA; 6 Department “Prevention, Rehabilitation and Social Medicine”, Deutsche Rentenversicherung (Germany), Bayreuth, Germany; 7 Department of Health Law, Policy and Management, Boston University School of Public Health, Boston Massachusetts, USA

**Keywords:** WD-FAB, WD-FAB-DE, Cultural adaptation, Forward-backward translation, Dual-panel translation, Item bank, Functional measurement, Disability measurement

## Abstract

**Background:**

The Work Disability Functional Assessment Battery (WD-FAB) was developed based on the International Classification of Functioning, Disability and Health (ICF) framework and administered using a computer adaptive test (CAT) to assess individuals’ functional status. The framework and assessment of functional status is especially pertinent in Germany as one factor in determining work disability. This paper reports on the translation and cultural adaptation of the WD-FAB from English (USA) to German (Germany).

**Methodology:**

A combination of forward-backward and dual-panel approaches was used to translate the WD-FAB into German. During the reconciliation process after forward and backward translations, a selection of items was discussed with the forward/backward certified translators, discussed with developers of the WD-FAB and selected for dual-panel discussion. A field test with rehabilitation patients was conducted to finalize the adaptation.

**Results:**

401 unique items from the original WD-FAB and version 3.0 (the majority of the items were the same across versions) were translated into German. After forward translation, the majority of the items were easily reconciled. Some items (identified by YF and TK) could not be easily reconciled and required additional examination. Based on the reasons for difficulties in forward translation, items were selected for the translation panel discussions (*n* = 69), discussed with developers (*n* = 49) and/or further discussed with the two forward translators (*n* = 18). After the first reconciliation and backward translation, 31 items were discussed with all translators again and 4 were again discussed with developers. 77 rehabilitation patients participated in the field test, 29 of whom provided feedback suggesting further revisions. Through the entire adaptation process, the items that were problematic involved those dealing with “anger” and “stress”. Furthermore, environmental differences between Germany and the USA limited the adaptation of some physical functioning items.

**Conclusions:**

Despite some differences in the cultural and structural context, we successfully adapted the WD-FAB item banks to German. Future validation of the measure is necessary to understand whether the items have similar psychometric measurement properties to those of the original WD-FAB.

## Background

Standardized and accurate methods for evaluating disability are essential for tracking individuals through treatment and rehabilitation, evaluating the effectiveness of interventions, and adjudicating social resources (e.g. disability pension). However, measuring work-related disability is a complex challenge given that an individual’s ability to work is influenced both by the individual’s functional level and the work environment. The World Health Organization (WHO) developed an international framework for describing, organizing, and measuring information on functional (dis)ability that closely aligns with the biopsychosocial model of health: the International Classification of Functioning, Disability and Health (ICF) [1, 2]. The ICF framework provides a uniform language across countries and health policy sectors by characterizing both the capability of the individual and their interaction with contextual factors. In Germany, the ICF model is embedded in social law and rehabilitation management programs [3, 4]. For example, the ICF is explicitly named in the Federal Law on Participation (Bundesteilhabegesetz; § 92 SGB V) to guide disability assessment by social security agencies as well as the guidelines of the Federal Joint Committee on medical rehabilitation services [5, 6].

**Fig. 1:**
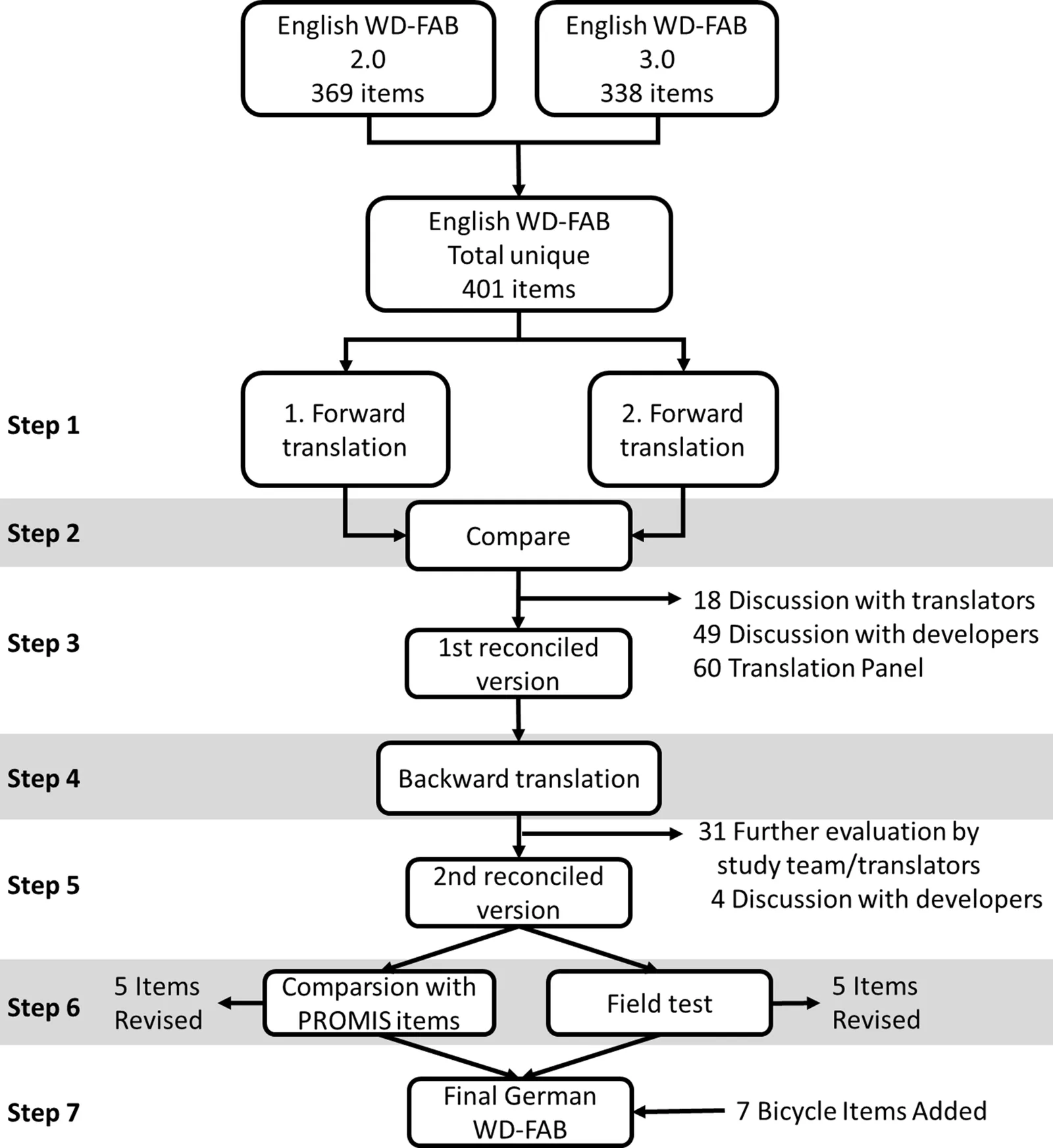
Adaptation process (English (American) to German (Germany)) combining forward-backward and dual-panel translation

**Fig. 2:**
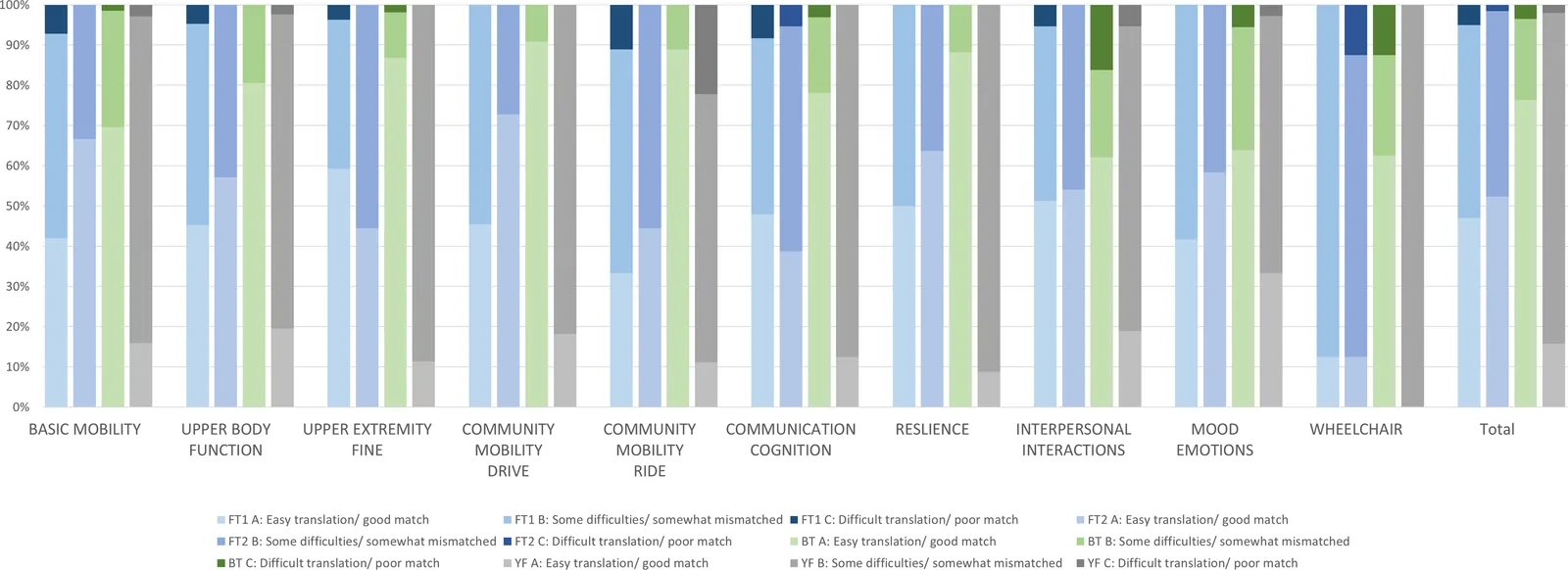
Translators’ and Author’s Ratings

The Work Disability Functional Assessment Battery (WD-FAB) was developed using item response theory (IRT) to support administration using computer adaptive testing (CAT). It integrates key components of the ICF conceptual framework to represent items at the activity-level of functional abilities [7]. It administers comprehensive item banks measuring 8 scales covering physical and mental functioning domains (plus a wheelchair-specific scale) using a CAT program to generate a comprehensive profile of work-related function [8, 9]. The WD-FAB development process was iterative and included literature review, focus groups, input from measurement and work disability experts, where the items were then psychometrically tested and calibrated in large samples of both Social Security Administration (SSA) disability claimants and general working age USA adults [8, 10, 11]. The original item banks has been replenished and refined since its original development [12, 13] and its item selection updated [14]. The item banks encompass over 300 items covering the dimensions of physical functioning (Basic Mobility, Upper Body Function, Fine Motor Function, and Community Mobility) and mental health functioning (Communication & Cognition, Self-Regulation, Mood & Emotions, and Resilience & Sociability [7, 9, 12, 13]. The combined item banks of WD-FAB versions 2.0 and 3.0 were adapted in this project.

Although the WD-FAB’s item banks are large, it is administered using CAT technology to minimize respondent burden while maintaining measurement accuracy [8, 9]. IRT estimates a respondent’s location on a latent scale “θ”, which represents a respondent’s ability, based on the pattern of responses to a set of questions. For example, responses to a set of items varying in levels of ‘difficulty’ on the WD-FAB Basic Mobility scale provides an estimation of an individual’s functional ability in this area. Using a computer-assisted process, an algorithm selects items based on responses to previous items until a respondent’s ability level is estimated with a certain level of confidence. Using this method, the WD-FAB can estimate respondent’s functional ability using 5 to 10 items per scale with precision similar to internal consistency reliability of ≥ 0.9 [12, 13].

The WD-FAB, a state-of-the-art instrument rigorously developed using the ICF framework, offers a globally relevant tool for adapting and testing across different international contexts. To develop a new self-reported instrument to measure work-related disability in Germany would be (1) resource intensive and (2) redundant since a highly reliable and valid technologically advanced tool already exists. It is therefore sensible to adopt the WD-FAB for use in Germany. A project to address a cross-cultural validation and translation of the WF-FAB within the context of the German social security system (Deutsche Rentenversicherung) has been undertaken with a goal to ensure equivalence of meaning (semantic equivalence [15]) between the source WD-FAB in English (USA) and the adapted WD-FAB-Deutsch (WD-FAB-DE) in German (Germany). The manuscript presents findings from the translation and adaptation phase of the project, specifically reporting on a hybrid process combining two recommended approaches to adapt the WD-FAB-DE. This is the first major step toward the overall goal of implementing the WD-FAB in Germany.

## Methods

We aimed to produce items which “ask” the same question in German, with consideration of the cultural context, as they do in English. While there is no strong evidence for preferred translation/adaptation approaches for self-reported health questionnaires [16–19], several guidelines exist [20–27]. From these guidelines, we selected two evidence-based approaches including forward-backward [21] and dual-panel [28]. The forward-backward approach is commonly used for health questionnaire translation and relies on a series of independent translations of the original language to the target language (forward translation) and then translating the reconciled target language version back into the original language (backward translation). However, even rigorous and independent forward-backward translations may not identify or resolve potential problems [29]. The dual-panel method involves one translation step (forward) by convening panels comprised of diverse samples of lay people, experts and certified translators to reach a consensus on translations for each item [28]. While both methods result in faithful translations [19, 30], the dual-panel method may produce translations preferred by lay participants [19]. However, applying the panel consensus method to all items of the WD-FAB item banks would be prohibitively time-consuming, expensive and not feasible for the scope of our project.

To adapt the WD-FAB, we used a combination of approaches by applying the forward-backward method to translate all items, and proceeding with the dual-panel method for a subset of items identified as problematic. Most guidelines for questionnaire translation employ several rounds of iterative revision in addition to formal translation including reconciliation, adjudication, and pretesting [20–27, 29, 31], which we adopted using a seven-step process (see Fig. 1). Owing to the large number of items translated, results are presented using representative examples of selected items.

At the inception of this project, WD-FAB version 2.0 was obtained from Boston University (369 items [12, 13, 32]). During the adaptation process (after the conclusion of step 1), we obtained an updated WD-FAB 3.0 from the National Institutes of Health (338 items [33]). Most items from the two versions overlap, but each also includes unique items. Because WD-FAB 2.0 already completed forward translation, we added all unique items from WD-FAB 3.0 to the adaptation project and therefore included all items from both versions (401 items).

### Translation/adaptation process

#### Step 1 forward translation

Two professional certified translators who are bilingual in English and German independently translated all items of the WD-FAB. The translators were instructed to consider the conceptual and cultural meaning of the items rather than word-for-word accuracy; to use vernacular German; to provide all possible alternatives when they could not choose a single translation; and to rate each item translation as.

A: meaning is semantically equivalent and the translation was straight-forward,
B: meaning is somewhat equivalent with some differences, or.
C: translation is not equivalent and/or item translated with difficulty.

#### Step 2 first adjudication and reconciliation of forward translations

Authors TK (native German speaker who is fluent in English) and YF (native American English speaker who is conversational in German), both scientists with expertise in patient-reported instruments as well as adapting health questionnaires into different languages, discussed and reconciled the two forward translations for all items. From this point, items were categorized into four groups:

1. reconciled,
2. items needing an alternate German translation, which were relayed back to forward translators,
3. items needing clarification of content, which were relayed to developers of the WD-FAB, and.
4. especially difficult translations that were selected for dual-panel discussion.

These classifications were not mutually exclusive, i.e. one item may belong to more than one group. As items that were not easily reconciled were flagged for further discussions, a third researcher was not required to reconcile disagreements.

Step 3a communication with forward translators: Telephone meetings were conducted between authors and forward translators to resolve problems with items belonging to group 2. Two conference calls were conducted to discuss inconsistencies and achieve satisfactory agreement between forward translators, TK, and YF. For the majority of the items, reconciliation could be reached by the two researchers and the forward translators. Items for which agreement could not be reached required additional translation evaluation. TK and YF identified whether discussion with developers and/or dual-panel translation were needed for each item.

#### Step 3b communication with developers

CM* and EM were two of the scientific leads during the development of the original WD-FAB [7, 8, 10–13, 32, 33], and supported the reconciliation of a set of items for which the conceptual definitions were not clear to the translation team. To ensure semantic equivalence, it was crucial to clarify the concepts of the original English items. Using telephone and video-conference communication, YF, CM* and EM discussed items identified as needing conceptual clarification including whether specific choices of German translations would be acceptable. Discussions with developers mainly took place before dual-panels were convened and before the backward translations. However, some discussions were also held after backward translations (step 4) and before the second reconciliation (step 5).

#### Step 3c dual translation panel

A translation panel, which consisted of native German speakers (*n* = 7) with differing levels of knowledge of self-reported health measures and the German rehabilitation care process, was convened across two meetings in early June of 2019. Panel members represented a range of ages (young adult to middle aged) and consisted of two men and five women. The panel also included diversity in terms of regions of Germany and leisure activities (e.g. one panel member who is a hobby soccer player offered the best translation for “kicking a ball”). YF and TK moderated the discussions to stay on topic, stay on schedule, and ensure all members of the panel had a chance to express their opinions.

Step 4 backward translation: After steps 2 and 3, the reconciled German version for all items were translated back into English by a translator (with over two decades of German/English translation experience) who is a native speaker of English and fluent in German. Similar to the forward translations, the backward translator was instructed to provide all possible alternatives when she could not choose one single translation, and to rank all translations as A, B, or C in the same way as described previously. Additionally, the translator was asked to give colloquial (language naturally spoken) rather than formal translations.

#### Step 5 second adjudication and reconciliation

The original English and reconciled German versions of the WD-FAB were compared. When necessary, all translators were contacted again to clarify cases when the deviations were large.

#### Step 6 field test

Best practices for instrument development include field testing an advanced version of the questionnaire prior to a larger validation study [34]. The field study involved participants completing WD-FAB items and then undergoing a cognitive debriefing interview with an experienced researcher. The interviews were used to evaluate the clarity of translations and identify wording that may be confusing or inappropriate. Accordingly, participants were asked to complete a set of WD-FAB-DE items after which the researcher lead a semi-structured discussion about difficulties in understanding items and response categories. Due to the large number of items, we administered a limited set of items (*n* ≈ 70) to each recruited field test participant to (1) ensure minimizing the time, effort, and burden, and (2) to feasibly conduct cognitive debriefing interviews. Rehabilitation patients were conveniently sampled from an outpatient rehabilitation and sport-therapy center in Greifswald, Germany. Additionally, wheelchair users were specifically recruited from a clinic specializing in treatment of patients with severe neurological conditions in Greifswald (BDH-Clinic).

Patients were given information letters and consented to participate in this project prior to each interview. Patients were informed that their participation was voluntary, they could withdraw their consent at any time during the interview, identifying information would not be collected and results would only be reported at the group level. Interviews with patients were approved by the research ethics review committee of the University Medicine Greifswald (approval identification BB113/19).

#### Step 7 final reconciliation

After backward translation, a small subset of items (*n* = 24) from the Basic Mobility (*n* = 3), Upper Body Function (*n* = 2), Fine Motor Function (*n* = 9) and Mood & Emotions (*n* = 10) scales were found to be shared with the Patient-Reported Outcome Measurement Information System (PROMIS [35–37]), This may be due to both instruments drawing from overlapping sources during development. Some of these PROMIS items have already been translated into German (Germany) by other research teams [38–40]. During Step 5, the item versions from this adaptation project were compared with the German PROMIS items and revised when deemed necessary (based on discussions among YSF, TK and SMM). Because the item overlap with PROMIS was discovered after field-testing already began, the comparison to PROMIS translations was conducted in parallel with step 6 (Fig. 1).

The WD-FAB-DE was finalized in a final reconciliation step after reviewing results of all field tests and relevant German PROMIS items.

## Results

### The WD-FAB instrument

The complete item banks of two versions of the WD-FAB were obtained from Boston University and the National Institutes of Health (WD-FAB 2.0 [12, 13, 32] and WD-FAB 3.0 [33]) which contain 401 unique items (Fig. 1, adaptation process).

### Forward translations and 1st reconciliation

The two forward translators generally found translations to be easy and clear, with the highest number of “C” rated translations assigned to items from the Community Mobility and Wheelchair scales (Fig. 2). Unclear conceptual definitions and a lack of equivalent daily activities in Germany accounted for the majority of reconciliation problems. Nonetheless, less than 10% of overall items were rated “C” by the translators.

After forward translations, 310 items were reconciled while 18 were further discussed with the forward translators, 49 were discussed with the WD-FAB developers (CM* and BM), and 60 items were flagged for discussion with the translation panel. These items overlap in that some items were flagged for multiple further evaluations. Table 1 gives examples of items that needed further evaluation.

**Table 1:** Example WD-FAB items needing further discussion with translators, developers and the translation panel

	English Original	Reconciled Forward Translation	Problem/decision	Translation resolution
Discussion with Translators	Please specify your level of agreement: If I make a mistake, I know I can deal with it.	Inwiefern stimmen Sie dieser Aussage zu: Wenn ich einen Fehler mache, weiß ich, dass ich **damit fertig werden kann**.	The two forward translations match, but during discussion the research team altered the forward translation of “damit fertig werden kann” (can cope with) to “damit umgehen kann” (can deal with). This revision was discussed with forward translators. Revision was approved by translators (with reservation from one)	Inwieweit trifft die folgende Aussage auf Sie zu: Wenn ich einen Fehler mache, weiß ich, dass ich **damit umgehen kann**.
	Please specify your level of agreement: I am able to do my work carefully.	Inwiefern stimmen Sie dieser Aussage zu: **Ich bin in der Lage**, meine Arbeit sorgfältig erledigen.	Research team wished to shorten “ich bin in der Lage” (able to) to “kann” (can). Translators did not approve this change, and the original language was used.	Inwieweit trifft die folgende Aussage auf Sie zu: **Ich bin in der Lage**, meine Arbeit sorgfältig zu erledigen.
	Please specify your level of agreement: I can keep up a conversation.	Inwiefern stimmen Sie dieser Aussage zu: Ich kann **eine Unterhaltung führen / ein Gespräch aufrechterhalten**.	Forward translations were meaningfully different: Discussion with translators settled on a slightly different final item. Final item highlighted “mit anderen unterhalten” (converse with others) and “Gespräch leicht aufrechterhalten” (easily maintain a conversation)	Wenn ich mich **mit anderen unterhalte**, kann ich das **Gespräch leicht aufrechterhalten**.
Discussed with Developers	Are you able to **keep yourself safe at home**?	Sind Sie in der Lage, zu **Hause für Ihre Sicherheit zu sorgen**?	Both translators found the concept “keep safe” to be ambiguous. Developers revealed that the item includes concepts (1) safety awareness (of external dangers) as well as (2) safely living independently. Cognitively, this item addresses both safety procedures and personal safety Final German translation was not altered as both concepts of safety are reasonably implied.	Sind Sie in der Lage, zu **Hause für Ihre Sicherheit zu sorgen**?
	Please specify your level of agreement: I am unable to think with all the **noise in my head**.	No reconciliation: one translator used **noise as in sounds** and the other **chaotic thoughts**	Noise can mean chaotic thoughts, or actual noise (e.g. tinnitus)? Developer confirmed that this item refers to chaotic thoughts. Final German version reflects “chaotic thoughts” meaning.	Inwiefern trifft folgende Aussage auf Sie zu: Mit all dem Durcheinander **in meinem Kopf kann ich keinen klaren Gedanken fassen**.
	Are you able to walk up a **steep slope**? For example on a hill.	Sind Sie in der Lage, einen steilen Hang (zum Beispiel auf einen **Hügel**) hinaufzugehen?	Translators were unsure of how steep the slope is (which matters for determine the appropriate German text) Developers confirmed the slope should be steeper than handicap ramp – or a “hill” in the American New England context Final German translation reflects this steepness.	Können Sie einen steilen Hang hinaufgehen (z.B. einen **Hügel**)?
	Are you able to lift a **2 L** soda bottle from the floor to a high shelf? A soda bottle = 3.5lbs/1.5 kg	Sind Sie in der Lage, eine **2-Liter** Sprudelflasche vom Boden auf ein hohes Regal zu heben? Eine Sprudelflasche = 1,5 Kilo	1.5 L is a more common beverage container size than 2 L. 1.5 L would also better correspond to 1.5 kg in weight. Revision to the text to include 1.5 L bottle was made with agreement of developers.	Können Sie eine volle **1**,**5-Liter** Sprudelflasche vom Fußboden auf ein hohes Regal heben?
Presented to translation panel	Are you able to **walk the aisles** of a grocery store using a shopping cart?	Sind Sie in der Lage, in einem Lebensmittelgeschäft mit einen **Einkaufswagen hin- und herzugehen**.	The translation “hin- und hergehen” (back and forth) implies that the individual is lost Solution panel suggested was adopted: “durch die Abteilungen gehen” (go through the sections/departments)	Sind Sie in der Lage, in einem Lebensmittelgeschäft mit einem Einkaufswagen **durch die Abteilungen zugehen**?
	Are you able to **kick** a ball?	Können Sie einen Ball **schießen/kicken**?	The translation for „kick“ is not satisfactory: there is no “generic” term for kick in German and neither forward translations captured the English meaning adequately. Panel solution (suggested by hobby soccer players) was adopted: “wegstoßen” (kick away).	Können Sie mit dem Fuß einen Ball **wegstoßen**?
	Are you able to make **small talk**?	Sind Sie in der Lage, **leichte Konversation zu machen**, sich beiläufig zu unterhalten?	„small talk“ is difficult to translate and translators suggested “leichte Konversation zu machen” (converse regarding light topics) is not satisfactory. Translation panel suggestion ”zu plaudern” (to chat) was adopted.	Sind Sie in der Lage, mit anderen **zu plaudern**?
Discussed with translators, developers and translation panel	How fast are you able to walk? • Faster than **those around me** • At a normal pace compared to **those around me** • At a slower pace than **those around me** • Unable to do • I don’t know	Wie schnell können Sie gehen? • Schneller als die **anderen Menschen um mich / andere in meiner Umgebung** • Im normal(en) Tempo **verglichen mit den anderen Menschen um mich herum / im Vergleich zu anderen in meiner Umgebung** • (**In einem**) langsameren Tempo als die **anderen** **Menschen um mich herum / als andere in meiner Umgebung** • Nicht dazu in der Lage • Weiß ich nicht	Translator discussion: the phrase “mit anderen Menschen um mich herum” was revised from both forward translations. Plausible use of the phrase Discussion with Developers: does the question distinguish between outside and indoor settings (which may affect the walking tempo)	Wie schnell können Sie gehen? • Schneller als **andere Menschen um mich herum** • Im normalen Tempo verglichen mit **andere Menschen um mich herum** • In einem langsameren Tempo als **andere Menschen um mich herum** • Dazu bin ich nicht in der Lage • Weiß ich nicht
	Are you able to work at floor level? For example **changing the face plate on an electric outlet**.	Können Sie auf Bodenhöhe arbeiten (z.B. um die **Abdeckung einer Steckdose auszutauschen** oder eine Kindersicherung anzubringen)?	Both forward translators raised the issue that the example is not a common activity in Germany. Developer approved to drop the electric outlet example, but to keep the work-relevant movement of working on a vertical service low to the floor. Researchers and translation Panel offered a different example: working with child safety plugs at floor level.	Können Sie auf Bodenhöhe arbeiten (z.B. um die Abdeckung einer Steckdose auszutauschen oder eine **Kindersicherung anzubringen**)?

Emphasis used for the section of text of interest, and not included in the items themselves

### Backward translation

The backward translator ratings were overall more positive than the forward translators with the largest percentage of “C” ratings given for the Interpersonal Interactions and Wheelchair scales (Fig. 2). The most problematic backward translations concerned the terms “angry” and “stress”, where the intensity was not clear in the back-translated English.

### Panel method

Sixty selected items were presented to the translation panel over two meetings. The panels generated new translation approaches and solutions that did not arise during forward translation or discussions with the translators. Pros and cons of alternate translations were discussed, and lay understandings of the concepts were prioritized. Two sessions were required (approximately 2 to 3 h) to resolve translations. Satisfactory solutions were found for nearly all (*n* = 56) items presented to the panel. The remaining four items were further discussed with the forward translators and reconciled considering all feedback from the panel discussions.

### Working with developers

A common issue during the adaptation process, both in discussion with translators and the panel members, was the conceptual definition of some of the items. Table 1 provides selected examples of items where there was ambiguity of meaning (more than one conceptual meaning could be applied to the wording of the item) or lack of clarity in concept of the original English. When the ambiguity permitted in the original English item (according to the developers) could not be retained in the German translation, the research team discussed the intended conceptual meaning of the item with the developers and selected a single interpretation for adaptation. In total, 49 items were discussed with the developers at all stages of the translation process. When necessary, revised translations based on discussions with the developers were taken back to the translators to refine wording. For most of these items, discussion with developers completed before the translation panel convened.

### Challenges with translation

Three main reoccurring issues were identified during the translation process: structural differences, the concept of “angry”, and the concept of “stress”.

Structural and environmental contextual differences were a source of difficulty during translation of items belonging to the physical functioning scales of the WD-FAB. This is of particular concern for items about the structural environment (such as door mechanisms: knobs versus latches) and daily activities (such as biking versus driving). Examples of items are provided in Table 2. A last set of items were problematic not because of translation but due to cultural/structural differences between Germany and the USA. For instance, an item asking about the ability to use twist tie closures on bread packages would not function well in Germany as bread is not packaged in this way. An option to preserve this motion would be to change the item to address electronics packaging, for which twist ties are commonly used in Germany. However, this involves a major reconstruction of the item. In such cases, the research team decided to maintain the original aim of semantic equivalence, and choose the translations that were as comparable to the original English as possible. Thus, some adapted items would address movements unlikely to be encountered in everyday life (e.g. turning a doorknob). These items were flagged as they may need major revision or be replaced by alternative items in the future.

**Table 2:** Example WD-FAB items with problems in translation due to cultural/structural differences between USA and Germany

English Original	Reconciled Forward Translation	Problem & Decision	Final Resolution
Are you able to close a **twist tie** on a bag of bread? Can you seal a bread bag with the fastening clip?	Können Sie den **Verschlussclip** an einem Beutel Brot schließen?	Bread is not packaged using twist ties in Germany. Although twist ties can be commonly found for electronic goods, it was the consensus of the German research team to majorly revise this item. The reconciled German translation refers to Verschlussclip (a closure clip) rather than twist tie, which is more often used for bread packaging. However, this changes the motor function of the original item.	Können Sie den **Verschlussclip** an einem Beutel Brot schließen?
Are you able to clean out a **closet**? Can you empty out a **wardrobe**?	Können Sie einen **Kleiderschrank** ausräumen?	It is not common in German living spaces to have built-in closets, and cleaning out a built-in closet requires different movements than a free-standing wardrobe. Furthermore, these two items have the same translation in German as there is no distinction between a freestanding wardrobe and a built-in closet. Final translation maintained the term “Kleiderschrank” for closet/wardrobe with a note that this is closer in meaning to the original English item concerning the wardrobe.	Können Sie einen **Kleiderschrank** ausräumen?
Are you able to **turn a door knob**? Can you **turn a doorknob**?	Können Sie einen **Türknauf drehen**?	The majority of doors in Germany operate via lever handle or a static (non-turning) knob. Changing the item to common German door mechanisms would change the motor function of this item. Final German version refers to turning a knob, but recognizing that this does not describe a common activity.	Können Sie einen **Türknauf drehen**?

Emphasis used for the section of text of interest, and not included in the items themselves

Linguistically, while some minor conceptual differences were observed (for instance, the difference in the way “kick” a ball can be generally understood in English versus the multiple ways to express kicking a ball in German), these conceptual differences were resolved during the translation panels and discussions with translators. However, two commonly observed differences that could not be easily resolved in translation were the concepts of “anger” and “stress”. The problems were most obvious in the back translations (Table 3), where various expressions of “stress” were interpreted to mean “stressful situations” whereas “anger” was translated to various degrees of emotion from “annoying” to “angry” based on conversations with the developers.

**Table 3:** Back translation inconsistencies of “Stress” and “Anger” items

	1st Reconciled German	English Original	English (Blind) Back Translation
**Example “Stress” Items**	Inwieweit trifft die folgende Aussage auf Sie zu: Ich kann mit **Stresssituationen** umgehen.	Please specify your level of agreement: I can handle **stressful situations**.	To what extent does the following statement apply to you: I can deal with **stressful situations**.
	Inwieweit trifft die folgende Aussage auf Sie zu: Es fällt mir schwer **Stresssituationen** durchzustehen.	Please specify your level of agreement: I find it difficult to get through **stressful events**.	To what extent does the following statement apply to you: It is difficult for me to get through **stressful situations**.
	Inwieweit trifft die folgende Aussage auf Sie zu: In **Stresssituationen** verliere ich regelmäßig die Kontrolle.	Please specify your level of agreement: **When I am stressed**, I find myself losing control.	To what extent does the following statement apply to you: In **stressful situations** I consistently lose control.
	Inwieweit trifft die folgende Aussage auf Sie zu: In **Stresssituationen** weiß ich nicht, was ich tun soll.	Please specify your level of agreement: **When I’m stressed**, I can’t figure out what to do.	To what extent does the following statement apply to you: I don’t know what to do in **stressful situations**.
**Example “Anger” Items**	Inwieweit trifft die folgende Aussage auf Sie zu: **Ich ärgere mich** oft, wenn man mir sagt, was ich tun soll.	Please specify your level of agreement: I often get **angry** when I’m told what to do.	To what extent does the following statement apply to you: It often **annoys me** when people tell me what to do. It usually annoys me when people tell me what to do.
	Inwieweit trifft die folgende Aussage auf Sie zu: Die Menschen wissen, dass **ich leicht ärgerlich werde**.	Please specify your level of agreement: People know that I **get angry** easily.	To what extent does the following statement apply to you: People know I am **easily annoyed**.
	Inwieweit trifft die folgende Aussage auf Sie zu: Ich werde manchmal handgreiflich, **wenn ich wütend bin**.	Please specify your level of agreement: I sometimes get physical **when I’m angry**.	To what extent does the following statement apply to you: I sometimes get violent **when I am angry**.
	In den letzten 7 Tagen wollte ich es jemandem heimzahlen, wenn ich mich über ihn **geärgert habe**.	In the past 7 days, I tried to get even when **I was angry** at someone.	In the last 7 days I wanted to pay someone back for **upsetting me**.

Emphasis used for the section of text of interest, and not included in the items themselves

### Field test

The field test consisted of rehabilitation patients (33 men, 44 women) with 6 additional wheelchair users sampled for the wheelchair specific items. More than one-third of the participants (*n* = 29) provided no additional comments for the items, and the remaining participants offered minor suggestions such as alternative wording for specific items: these were all deemed non-systematic and minor. One WD-FAB-DE item was modified according to the field test results: “Können Sie einen vollen Einkaufswagen ins Auto umladen?” was revised to “Können Sie **den Inhalt** eines vollen Einkaufswagens ins Auto umladen?” (bold added to specify the revised text; English original: “Are you able to unload a full grocery cart into a car?”). Wheelchair items were found to be more problematic in the field test for which the majority of the feedback addressed the lack of specificity of the environment. Four of the 8 wheelchair items were modified using feedback from field test participants.

### Bicycle items

During cross-cultural adaptation, the research team identified a need to add bicycle items to the Community Mobility scale. This is due to bicycling being commonplace in Germany for daily commuting across all ages and regions. The original USA version of the WD-FAB (Community Mobility scale) focused on automobile and public transportation. Therefore, the research team adapted 7 of the driving items from the Community Mobility scale to bicycle travel (Table 4). The next phase of this project includes empirical validation of these items.

**Table 4:** Example of created biking items for the WD-FAB-DE dimension Community Mobility

English “Drive” Items	Reconciled German “Drive” Items	Created Biking Items
Are you able to drive in heavy traffic?	Können Sie bei dichtem Verkehr **Auto fahren**?	Können Sie bei dichtem Verkehr **Fahrrad fahren**?
Are you able to drive in your own neighborhood?	Sind Sie in der Lage, in Ihrer näheren Umgebung **Auto zu fahren**?	Sind Sie in der Lage, in Ihrer näheren Umgebung mit dem **Fahrrad zu fahren**?
Please specify your level of agreement: I am only comfortable driving short distances.	Inwieweit trifft die folgende Aussage auf Sie zu: **Mit dem Auto** fahre ich lieber nur kurze Strecken.	Inwieweit trifft die folgende Aussage auf Sie zu: **Mit dem Fahrrad** fahre ich lieber nur kurze Strecken.
Please specify your level of agreement: I can drive to a local store and back home on my own.	Inwieweit trifft die folgende Aussage auf Sie zu: Ich kann alleine **mit dem Auto** zum nächsten Geschäft und wieder nach Hause fahren.	Inwieweit trifft die folgende Aussage auf Sie zu: Ich kann alleine **mit dem Fahrrad** zum nächsten Geschäft und wieder nach Hause fahren.

Emphasis used for the section of text of interest, and not included in the items themselves

### PROMIS items

The 24 items shared between the WD-FAB and PROMIS were not identical: the WD-FAB items often included examples of the activities/function whereas the PROMIS items did not. After further review, modifications were made to 5 WD-FAB-DE items (see Table 5).

**Table 5:** Examples of reconciling WD-FAB and PROMIS Germany adaptations

PROMIS Original English	WD-FAB Original English	PROMIS Official German	WD-FAB reconciled German (Emphasis on differences from PROMIS German version)	Decision
Are you able to stand up from an armless straight chair?	Are you able to stand up from an armless, straight chair? Hint: Without holding on to anything.	Können Sie von einem Stuhl ohne Armlehnen aufstehen?	Können Sie von einem **normalen** Stuhl ohne Armlehnen aufstehen, **ohne sich abzustützen**?	Prefer our adaptation
Are you able to open a can with a hand can opener?	Are you able to open a can with a hand can opener?	Können Sie eine Dose mit einem Dosenöffner öffnen?	Können Sie mit einem **Handdosenöffner eine Dose** öffnen?	Prefer the PROMIS adaptation
Are you able to cut a piece of paper with scissors?	Are you able to cut a piece of paper with scissors?	Können Sie ein Stück Papier mit einer Schere schneiden?	Können Sie ein **Blatt Papier** mit einer Schere schneiden?	Prefer our adaptation
In the past 7 days, I felt that I had nothing to look forward to.	In the past 7 days, I felt that I had nothing to look forward to.	Ich hatte das Gefühl, dass ich mich auf nichts freuen kann.	In den letzten 7 Tagen hatte ich das Gefühl, mich auf nichts freuen zu **können**.	Prefer our adaptation
In the past 7 days, I felt that nothing was interesting.	In the past 7 days, I felt that nothing was interesting.	Ich hatte an nichts Interesse.	In den letzten 7 Tagen **hatte ich das Gefühl**,** dass für mich nichts interessant war.**	Further revised
In the past 7 days, I felt that nothing could cheer me up.	In the past 7 days, I felt that nothing could cheer me up.	Ich hatte das Gefühl, dass mich nichts aufheitern kann.	In den letzten 7 Tagen hatte ich das Gefühl, dass mich nichts aufheitern **könnte.**	Prefer our adaptation
In the past 7 days, I felt emotionally exhausted.	In the past 7 days, I felt emotionally exhausted.	Ich fühlte mich seelisch erschöpft.	In den letzten 7 Tagen fühlte ich mich **emotional ausgelaugt**.	Further revised

Emphasis used for the section of text of interest, and not included in the items themselves

### Final reconciliation

A final reconciliation took place after the field test was completed and German PROMIS items were assessed.

## Discussion

We successfully translated and culturally adapted the WD-FAB item banks from English (USA) to German (Germany), preserving compatibility with the original USA-calibrated CAT system. The challenge of cross-cultural adaptation of a large item bank is feasibility in terms of time and cost. Furthermore, a rigorous translation requires continuous contact with translators and developers through several rounds of adjudication to ensure adherence to semantic equivalence, as well as identifying issues in translation that may need further adaptation. A hybrid approach ensured feasibility with added benefit compared to either method (forward-backward and dual-panel) individually applied. The forward-backward method is sufficient for the majority of items, and given the large number of items in the item banks, can be completed within a reasonable time and budget. For many of the items for which problems arose during forward translation, a translation panel was not needed: rather it was more informative to discuss and clarify conceptual questions with the developers and forward translators. The translation panel was convened for a short-list of items that would benefit most from a panel discussion, ensuring that the panels were conducted within an acceptable number of hours. With continuous feedback from various perspectives, we produced the German WD-FAB-DE, which is hypothesized to conceptually measure comparable latent constructs as the original English version. It is possible that this hybrid approach can be further streamlined: future empirical research can clarify the necessary steps of forward-backward and dual panel approaches to ensure semantic equivalence.

This adaptation was conducted with clear and frequent communication with all translators, the translation panel and the developers of the original instrument. Collaborative relationships across all parties may need to be present for any successful adaptation, especially if components (e.g. backward translation) are excluded. Our research team is experienced, with translators having prior experience translating self-rated health instruments and being able to anticipate potential problems. Additionally, there is high value that the primary researchers leading the adaptation project and the adjudication process at all stages (YF and TK) are native speakers and have lived cultural experiences of the original and target languages/countries, respectively.

This hybrid approach is perhaps most useful for adaptations for which the original and target languages/settings do not differ substantially in terms of cultural understandings of the dimensions of interest as well as the social and contextual environments. For this project, Germany and America are both western OECD (Organisation for Economic Co-operation and Development) countries with relatively comparable concepts of physical and mental functioning. The hybrid method may be more difficult for cross-cultural adaptations for which the original and target cultures differ more substantively [41, 42], and a larger proportion of the items need to be presented to the translation panel and/or a larger proportion of items need substantive revisions between forward-backward and dual-panel translation.

Herdman et al. (1997)’s seminal overview of the concept of “equivalence” in translation and adaptation of self-reported health questionnaires emphasized the cultural component and the importance of contextual equivalence [15]. They recommended adopting a universalist approach focused on preserving concepts that maintain comparable meaning across cultural contexts. A major advantage of adapting the WD-FAB is its foundation within the ICF conceptual framework, which is widely recognized and applied in both USA and Germany. Therefore, the research team assumed a level of conceptual equivalence across functional measurements between the USA and Germany. We used a universalist orientation to ground our approach, with a focus on semantic equivalence, assuming a relevant level of conceptual equivalence due to the shared ICF framework. Despite the shared concept of functional status, we found differences in daily activities led to difficulty translating some items. Examples include Germans not typically using twist-tie closures for bread packaging and most German homes not having built-in closets (see Table 2). Additionally, during the adaptation process, it was determined that the contextual differences around the Community Mobility scale were problematic. While we created additional “biking” items to address this problem, the Community Mobility scale needs further future research into modifications for the German context.

For a small set of items, particularly for the physical function scales, there were cultural/structural issues with adaptation. The WD-FAB uses daily activities to assess function, and those same daily activities may involve different specific movements in Germany than in the USA. For instance, turning a doorknob (USA) involves a different movement than pressing down on a door lever (Germany). Problems with cultural context when adapting items, especially those measuring physical functioning, have also been found for the Korean adaptation of the PROMIS measure [43]. Our team decided to preserve the physical activity items in the German WD-FAB-DE so that the meaning of the original English is not changed. To capture the same movement, the item may need to be re-written rather than simply revised or adapted. For this reason, it is crucial to validate the item banks and calibrate the instrument in a German population using rigorous psychometric methods with Rasch-based Item Response Theory. An advantage of using a CAT instrument based on IRT calibration is that we can replenish its item banks [12, 13, 44], making the measure more adaptable than static measures. In the future, items that differ in contextual meaning but capture the intended functional constructs could be added to the WD-FAB item banks through ongoing replenishments and recalibration.

Although previous studies have not identified substantial differences between (1) forward plus backward translation alone [19], and (2) forward-backward translation versus dual-panel approaches [19, 30], our field testing demonstrated that these processes can still provide valuable information leading to meaningful item revisions. In particular, the field test was especially important for the wheelchair items, revealing that some contextual information and terminology remained suboptimal after the translation process. This finding supports the conclusion of Walde and Vollm (2023) that testing with the intended user population is essential in cross-cultural adaptation (2023) that testing with the intended user population is essential in cross-cultural adaptation [29].

The main limitation of this manuscript is that it reports only on the translation and adaptation phase of the project, and therefore focuses on content validity (the rigorous adaptation process) and face validity (translation panel and field testing). Many other aspects of validity such as factorial, convergent, divergent and discriminant validity were not addressed. Furthermore, content validity was not included in the field test due to the large number of items each patient evaluated. Similarly, test-retest reliability and responsiveness to change were not included. Currently, work to validate the instrument in the German population and provide German-calibrated IRT weights for the WD-FAB-DE is ongoing. All items from this version of the WD-FAB-DE will be included and further revisions/exclusions will be based on item performance. Additionally, the Community Mobility and Wheelchair scales need further research into determining appropriate items for the German context: this will involve communications with patients and professional experts, as well as a review of German (or Western European) specific instruments.

## Conclusion

The WD-FAB item banks were successfully translated and culturally adapted for use in Germany. The combined use of forward-backward and dual panel translation approaches was valuable for adapting a large CAT-based item bank while preserving semantic and conceptual equivalence across languages and contexts. Field testing further supported the acceptability and clarity of the preliminary WD-FAB-DE. Future work will focus on psychometric validation, calibration in German populations, and continued refinement of culturally relevant items within the WD-FAB framework.

## Data Availability

The only data available from this project are the full item-bank adaptation tables and noted interview responses. Due to the items still needing calibration and possibly used in the decision-making process for claims adjudication in Germany, the item-bank cannot be fully published. We provide example items in the manuscript tables which illustrate various components of the adaptation process. The debriefing interview notes can be made available upon request.

## Abbreviations

CAT: Computer Adaptive Testing
DE: Deutschland/Germany
ICF: International Classification of Functioning, Disability and Health
IRT: Item Response Theory
PROMIS: Patient-Reported Outcome Measurement Information System
SSA: Social Security Administration
USA: United States of America
WD-FAB: Work Disability Functional Assessment Battery
WD-FAB-DE: Work Disability Functional Assessment Battery Adapted to German (Germany)
